# Associated Urogenital Injuries Following Pelvic Trauma in Pediatrics: A 15-Year Single-Center Retrospective Study

**DOI:** 10.7759/cureus.107462

**Published:** 2026-04-21

**Authors:** Colin Van Wagoner, Talon Shumway, Arthur Saroyan, Layth Saleh, Sazid Hasan, Alexandria Chrumka, Ehab Saleh

**Affiliations:** 1 Orthopedic Surgery, Oakland University William Beaumont School of Medicine, Auburn Hills, USA; 2 College of Literature, Science, and the Arts, University of Michigan, Ann Arbor, USA; 3 Orthopedic Surgery, University of Toledo, Toledo, USA; 4 Orthopedic Surgery, University of Wisconsin, Madison, USA; 5 Orthopedics, Beaumont Hospital, Royal Oak, USA

**Keywords:** pediatric trauma, pelvic ring fracture, retrospective studies, urethral injury, urogenital trauma

## Abstract

Objectives: The association between pelvic trauma and urogenital injuries in adults is well-established, whereas pediatric literature is forthcoming. This study aimed to determine the association between pelvic trauma and urogenital injuries in pediatric patients, including incidence, injury patterns, diagnosis, and treatment. We hypothesized that pediatric patients would demonstrate decreased rates of urogenital injuries associated with pelvic trauma compared to adults.

Methods: This was a single-center, retrospective chart review. Patients aged 0-16 years admitted for pelvic fracture, pelvic dislocation, proximal femur fracture, and injury of urinary and pelvic organs over a 15-year period were included. Primary variables included fracture diagnosis, urogenital injury, treatment modalities, and mechanism of injury. Secondary outcomes included patient demographics, length of hospital and intensive care unit (ICU) stay, imaging, and Foley placement. ANOVA and chi-square analysis were used for statistical analysis.

Results: A total of 371 patient charts were reviewed. Following additional exclusion criteria, 303 patients were compared for analysis. Patients were grouped based on primary fracture diagnosis: pelvic ring fracture (49.2%, n = 149), femur fracture (42.2%, n = 128), and acetabular fracture/hip dislocation (8.6%, n = 26). There was no associated urogenital injury in 81.9% (n = 122) of the pelvic ring fracture group, 90.6% (n = 116) of the femur fracture group, and 100% (n = 26) of the acetabular fracture/hip dislocation group (p = 0.0690).

Conclusions: Pelvic trauma with associated urogenital injury is less likely in pediatric patients than in adults. When present, it is more commonly associated with the male gender and increased pelvic fracture severity.

Level of evidence: Level III - retrospective chart review.

## Introduction

The link between pelvic fractures and urogenital injuries is well-documented in adults but less understood in children. Pelvic fractures are relatively uncommon, representing a small percentage of all fractures in adults, with an even lower incidence in pediatric patients [1,2]. The incidence of these injuries is significantly increased in high-energy, blunt-trauma events like motor vehicle accidents or severe falls, reaching as high as 25% following such trauma [1,3,4]. Among pelvic fractures, genitourinary insults are most commonly observed in anterior arch pelvic ring injuries such as displaced pubic bone fractures and pubic symphysis diastasis [2,5,6]. Nearly a quarter of these fractures present with a urinary tract lesion, occurring more frequently in men [5]. Individuals with urogenital injury have increased rates of urinary incontinence, sexual dysfunction, strictures, and chronic pelvic pain [5,7,8]. Such sequelae can significantly impact patients’ quality of life [9]. Additionally, patients with lesions of the urogenital system have a higher risk of mortality [5]. Early identification and appropriate management are vital in preventing long-term morbidity and improving quality of life outcomes [7,9].

The existing literature regarding pelvic fractures and urogenital injury specifically in pediatric populations remains limited [2]. Previous pediatric-centered studies demonstrate conflicting data surrounding incidence rates of urogenital injury associated with pelvic trauma [10]. The primary purpose of this study was to assess the relationship between pelvic trauma and urogenital injuries in pediatrics. Secondary aims included assessing the incidence of pelvic trauma, injury patterns, mechanism of injury, treatment approaches, imaging techniques, and other factors related to the hospital stay. We hypothesized that pediatric patients would demonstrate decreased rates of urogenital injuries associated with pelvic trauma compared to adults.

This article was previously presented as a poster at the Great Lakes Pediatrics Research Day in March 2025, as well as the Mid-America Orthopaedic Association Annual Meeting in April 2025.

## Materials and methods

This study was a single-center, retrospective chart review performed at a level 1 trauma center. Institutional Review Board approval was granted for project completion. The electronic medical record was queried for patient encounters between January 2007 and January 2022. Inclusion criteria included patients aged 0-16 years, inpatient admission, and admission diagnosis codes for pelvic fracture, pelvic dislocation, proximal femur fracture, and injury of urinary and pelvic organs.

Data collected for each encounter included patient demographics, admission date, discharge date, fracture diagnosis, urogenital injury diagnosis, mechanism of injury (MOI), and treatment modality for both bony and urogenital injuries. Additional information collected included fracture classification using both the Tile classification and Torode and Zieg (Torode) classification systems, imaging modality, hospital and intensive care unit (ICU) length of stay, and Foley catheter placement [11-13].

The initial search produced 371 patient charts. Following review, additional charts were excluded due to restricted access, insufficient information, no pelvic fracture or urogenital injury, or a slipped capital femoral epiphysis (SCFE) or coccyx fracture diagnosis, leaving 303 patients for statistical analysis.

The data were first analyzed generally, and then separated into three groups for comparison: pelvic ring fractures, femur fractures, and acetabular fractures or hip dislocations. Statistical tests included an ANOVA F-test for quantitative data and chi-square analysis for categorical data. The statistical test used was denoted with a numerical superscript over the associated P-value. P-values of less than 0.05 were considered statistically significant.

## Results

**Figure 1:**
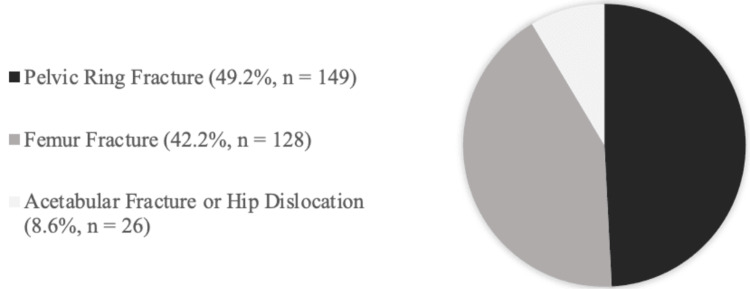
Pie chart representing the breakdown of primary fracture diagnosis at the time of admission within the study cohort.

A total of 303 patients were included for statistical analysis and comparison. As seen in Table 1, the mean age of the cohort was 11.0 years, with the majority of patients identifying as predominantly male and Caucasian. When sub-classified by primary fracture diagnosis, 91.4% (n = 277) of patients were admitted for either a pelvic ring or femur fracture (Figure 1).

**Table 1:** Demographic data of the study cohort, including age at admission, sex, and race, with mean (standard deviation), median, and range values provided for continuous data and raw values and percentages provided for categorical data.

Age at admission	
N	303
Mean (SD)	11.0 (4.6)
Median	13
Range	0-16
Sex, n (%)	
Female	102 (33.7%)
Male	201 (66.3%)
Race, n (%)	
American Indian or Alaska Native	2 (0.7%)
Asian	5 (1.7%)
Black or African American	61 (20.1%)
Other	24 (7.9%)
Prefer not to answer	12 (4.0%)
Unavailable	13 (4.3%)
White or Caucasian	186 (61.4%)

There were significant differences in the demographic characteristics between fracture diagnosis groups (Table 2). Patients experiencing pelvic ring fractures were the oldest, being one year older than the acetabular fracture group and nearly four years older than the femur fracture group (p < 0.0001). Additionally, sex distribution differed significantly across groups (χ²(2) = 9.0, p = 0.011.5, Cramér’s V = 0.17), with a male predominance observed in the femur fracture and acetabular fracture groups (approximately 3:1 and 4:1, respectively), compared to a more balanced sex distribution in the pelvic ring fracture group.

**Table 2:** Demographic data of the study cohort according to primary fracture diagnosis, with mean (standard deviation), median, and range values provided for continuous data and raw values and percentages provided for categorical data. ^1^ ANOVA F-test p-value. ^2^ Chi-square p-value.

	Pelvic ring fracture	Femur fracture	Acetabular fracture or hip dislocation	P-value
	N = 149	N = 128	N = 26	
Age at admission				<0.0001^1^
N	149	128	26	
Mean (SD)	12.7 (3.4)	8.9 (5.0)	11.7 (4.5)	
Median	14	11	13	
Range	2-16	0-16	0-16	
Sex, n (%)				0.0115^2^
Female	62 (41.6%)	35 (27.3%)	5 (19.2%)	
Male	87 (58.4%)	93 (72.7%)	21 (80.8%)	
Race, n (%)				0.0203^2^
American Indian or Alaska Native	2 (1.3%)	0 (0.0%)	0 (0.0%)	
Asian	2 (1.3%)	3 (2.3%)	0 (0.0%)	
Black or African American	30 (20.1%)	25 (19.5%)	6 (23.1%)	
Other	9 (6.0%)	13 (10.2%)	2 (7.7%)	
Prefer not to answer	8 (5.4%)	1 (0.8%)	3 (11.5%)	
Unavailable	7 (4.7%)	2 (1.6%)	4 (15.4%)	
White or Caucasian	91 (61.1%)	84 (65.6%)	11 (42.3%)	

Patients admitted for pelvic ring fractures were classified with both the Tile and Torode classification systems (Table 3). The three most common Tile classifications were A1 (46.9%, n = 69), A2 (23.1%, n = 34), and B2 (15.0%, n = 22). The three most common Torode classifications were I (46.3%, n = 68), IIIB (20.4%, n = 30), and IIIA (15.0%, n = 22).

**Table 3:** Tile and Torode classifications of fractures within the pelvic ring fracture group (n = 149) reported as raw values and percentages.

Tile classification	n (%)
A1	69 (46.9%)
A2	34 (23.1%)
A3	10 (6.8%)
B1	1 (0.7%)
B2	22 (15.0%)
B3	3 (2.0%)
C1	1 (0.7%)
C2	7 (4.8%)
Missing	2
Torode classification	
I	68 (46.3%)
II	11 (7.5%)
IIIA	22 (15.0%)
IIIB	30 (20.4%)
IV	16 (10.9%)
Missing	2

The remaining statistical variables presented were compared between fracture diagnosis groups. For each patient, the MOI was classified as either a fall, motor vehicle collision (MVC), blunt trauma, or other. As seen in Table 4, the most common MOI for each group showed pelvic ring fractures were predominantly caused by MVCs, femur fractures by falls, and acetabular fractures and hip dislocations primarily resulted from an MOI classified as "other" (χ²(6) = 45.17, p < 0.0001, Cramér’s V = 0.27). Overall, there was a significant number of patients within the cohort whose MOI was classified as "other".

**Table 4:** A comparison of the mechanism of injury between patients within each fracture diagnosis group, represented as raw values and percentages. ^1^ Chi-square p-value.

	Pelvic ring fracture	Femur fracture	Acetabular fracture or hip dislocation		P-value
	N = 149	N = 128	N = 26		
Injury mechanism, n (%)				<0.0001^1^	
Fall	30 (20.1%)	64 (50.0%)	6 (23.1%)		
Motor vehicle collision (MVC)	60 (40.3%)	12 (9.4%)	8 (30.8%)		
Blunt trauma	3 (2.0%)	4 (3.1%)	1 (3.8%)		
Other	56 (37.6%)	48 (37.5%)	11 (42.3%)		

The presence and type of associated urogenital injuries are reported in Table 5. Most patients did not experience associated urogenital injuries, with results showing no urogenital injury in 81.9% (n = 122), 90.6% (n = 116), and 100.0% (n = 26) of the pelvic ring fracture group, femur fracture group, and acetabular fracture group, respectively (χ²(10) ≈ 17.5, p = 0.069, Cramér’s V = 0.17). A total of three patients experienced urethral or bladder injury, all of whom were in the pelvic ring fracture group, accounting for 2% of that patient population. Urogenital injuries within the pelvic ring fracture group were further analyzed based on fracture classification (Tables 6-9). Based on the Tile classification system, urogenital injuries were associated with a wide range of fracture classifications but did show significant differences between groups (χ²(10) ≈ 29.6, p < 0.0001, Cramér’s V = 0.40). Analysis of the Torode classification indicated that patients with a group IV fracture had the highest association with urogenital injuries (56.2% injury rate) and accounted for the greatest number of urogenital injuries in a single group (n = 9) (χ²(20) ≈ 53, p < 0.0001, Cramér’s V = 0.30). The three patients experiencing urethral or bladder injuries had Tile fracture classifications of A2, B2, and C2, respectively, and had Torode fracture classifications of II (n = 1) and IV (n = 2).

**Table 5:** A comparison of the incidence and types of concomitant urogenital injuries according to fracture diagnosis groups, reported as raw values and percentages.​​​​​​​ ^1^ Chi-square p-value.

	Pelvic ring fracture	Femur fracture	Acetabular fracture or hip dislocation	P-value
	N = 149	N = 128	N = 26	
Urogenital injury, n (%)				0.0690^1^
None	122 (81.9%)	116 (90.6%)	26 (100.0%)	
Hematuria	10 (6.7%)	1 (0.8%)	0 (0.0%)	
Superficial injury	5 (3.4%)	1 (0.8%)	0 (0.0%)	
Urethral/bladder injury	3 (2.0%)	0 (0.0%)	0 (0.0%)	
Urinary retention or incontinence	8 (5.4%)	8 (6.3%)	0 (0.0%)	
Other	1 (0.7%)	2 (1.6%)	0 (0.0%)	

**Table 6:** A comparison of the incidence and types of concomitant urogenital injuries according to Tile A fracture classification for patients in the pelvic fracture group, represented as raw values and percentages.​​​​​​​ ^1^ Chi-square p-value.

	A1 (N = 69)	A2 (N = 34)	A3 (N = 10)	P-value
Urogenital injury, n (%)				<0.0001^1^
None	68 (98.6%)	22 (64.7%)	9 (90.0%)	
Hematuria	0 (0.0%)	4 (11.8%)	1 (10.0%)	
Superficial injury	1 (1.4%)	0 (0.0%)	0 (0.0%)	
Urethral/bladder injury	0 (0.0%)	1 (2.9%)	0 (0.0%)	
Urinary retention or incontinence	0 (0.0%)	6 (17.6%)	0 (0.0%)	
Other	0 (0.0%)	1 (2.9%)	0 (0.0%)	

**Table 7:** A comparison of the incidence and types of concomitant urogenital injuries according to Tile B fracture classification for patients in the pelvic fracture group, represented as raw values and percentages.​​​​​​​ ^1^ Chi-square p-value.

	B1 (N = 1)	B2 (N = 22)	B3 (N = 3)	P-value
Urogenital injury, n (%)				<0.0001^1^
None	0 (0.0%)	15 (68.2%)	1 (33.3%)	
Hematuria	0 (0.0%)	4 (18.2%)	1 (33.3%)	
Superficial injury	1 (100.0%)	0 (0.0%)	1 (33.3%)	
Urethral/bladder injury	0 (0.0%)	1 (4.5%)	0 (0.0%)	
Urinary retention or incontinence	0 (0.0%)	2 (9.1%)	0 (0.0%)	
Other	0 (0.0%)	0 (0.0%)	0 (0.0%)	

**Table 8:** A comparison of the incidence and types of concomitant urogenital injuries according to Tile C fracture classification for patients in the pelvic fracture group, represented as raw values and percentages. ^1^ Chi-square p-value.

	C1 (N = 1)	C2 (N = 7)	P-value
Urogenital injury, n (%)			<0.0001^1^
None	0 (0.0%)	5 (71.4%)	
Hematuria	0 (0.0%)	0 (0.0%)	
Superficial injury	1 (100.0%)	1 (14.3%)	
Urethral/bladder injury	0 (0.0%)	1 (14.3%)	
Urinary retention or incontinence	0 (0.0%)	0 (0.0%)	
Other	0 (0.0%)	0 (0.0%)	

**Table 9:** A comparison of the incidence and types of concomitant urogenital injuries according to Torode fracture classification for patients in the pelvic fracture group, represented as raw values. ^1^ Chi-square p-value.

	I (N = 68)	II (N = 11)	IIIA (N = 22)	IIIB (N = 30)		IV (N = 16)	P-value
Urogenital injury, n (%)							<0.0001^1^
None	67 (98.5%)	5 (45.5%)	17 (77.3%)	24 (80.0%)		7 (43.8%)	
Hematuria	0 (0.0%)	2 (18.2%)	2 (9.1%)	3 (10.0%)		3 (18.8%)	
Superficial injury	1 (1.5%)	0 (0.0%)	0 (0.0%)	1 (3.3%)		3 (18.8%)	
Urethral/bladder injury	0 (0.0%)	1 (9.1%)	0 (0.0%)	0 (0.0%)		2 (12.5%)	
Urinary retention or incontinence	0 (0.0%)	3 (27.3%)	2 (9.1%)	2 (6.7%)		1 (6.3%)	
Other	0 (0.0%)	0 (0.0%)	1 (4.5%)	0 (0.0%)		0 (0.0%)	

Treatment modalities were reported for both bony and urogenital injuries. To separate the location of injury, the treatment of fractures was classified as either pelvis (operative versus non-operative) or femur (operative versus non-operative). Table 10 shows significant differences in treatment modalities between groups (χ²(8) ≈ 88, p < 0.0001, Cramér’s V = 0.38), in which most pelvic ring fractures were treated non-operatively, whereas femoral and acetabular fractures showed a greater balance between operative and non-operative approaches. Regarding the treatment of urogenital injuries between groups, non-operative treatment was most common, but there was a significant difference (χ²(4) ≈ 11.3, p = 0.0235, Cramér’s V = 0.14) in that all five patients treated with any intervention other than a Foley catheter had a pelvic ring fracture. The treatment of urogenital injuries was also analyzed according to the Tile (Tables 11-13) and Torode classifications (Table 14). Based on these classifications, significant differences were noted (χ²(8) ≈ 42, p < 0.0001, Cramér’s V = 0.38), indicating that a Tile classification of C2 or Torode classification of IV was associated with the highest rates of interventional treatment for urogenital injuries.

**Table 10:** A comparison of treatment modalities for both bony and urogenital injuries, separated according to primary fracture diagnosis, represented as raw values and percentages. ^1^ Chi-square p-value. A comparison of treatment modalities for both bony and urogenital injuries, separated according to primary fracture diagnosis, represented as raw values and percentages.

	Pelvic ring fracture	Femur fracture	Acetabular fracture or hip dislocation	P-value
	N = 149	N = 128	N = 26	
Treatment bone, n (%)				<0.0001^1^
Pelvis - Operative treatment	16 (10.7%)	1 (0.8%)	11 (42.3%)	
Pelvis - Non-operative treatment	133 (89.3%)	0 (0.0%)	15 (57.7%)	
Femur - Operative treatment	0 (0.0%)	73 (57.5%)	0 (0.0%)	
Femur - Non-operative treatment	0 (0.0%)	53 (41.7%)	0 (0.0%)	
Missing	0	1	0	
Treatment urogenital, n (%)				0.0235^1^
No injury present	122 (81.9%)	116 (90.6%)	26 (100.0%)	
Non-operative treatment	22 (14.8%)	12 (9.4%)	0 (0.0%)	
Any intervention other than Foley	5 (3.4%)	0 (0.0%)	0 (0.0%)	

**Table 11:** A comparison of treatment modalities for urogenital injuries according to Tile fracture classification A for patients in the pelvic fracture group, represented as raw values and percentages. ^1^ Chi-square p-value.

	A1 (N = 69)	A2 (N = 34)	A3 (N = 10)	P-value
Treatment urogenital, n (%)				<0.0001^1^
No injury present	68 (98.6%)	22 (64.7%)	9 (90.0%)	
Non-operative treatment	1 (1.4%)	11 (32.4%)	1 (10.0%)	
Any intervention other than Foley	0 (0.0%)	1 (2.9%)	0 (0.0%)	

**Table 12:** A comparison of treatment modalities for urogenital injuries according to Tile fracture classification B for patients in the pelvic fracture group, represented as raw values and percentages.​​​​​​​ ^1^ Chi-square p-value.

	B1 (N = 1)	B2 (N = 22)	B3 (N = 3)	P-value
Treatment urogenital, n (%)				<0.0001^1^
No injury present	0 (0.0%)	15 (68.2%)	1 (33.3%)	
Non-operative treatment	1 (100.0%)	6 (27.3%)	1 (33.3%)	
Any intervention other than Foley	0 (0.0%)	1 (4.5%)	1 (33.3%)	

**Table 13:** A comparison of treatment modalities for urogenital injuries according to Tile fracture classification C for patients in the pelvic fracture group, represented as raw values and percentages.​​​​​​​ ^1^ Chi-square p-value.

	C1 (N = 1)	C2 (N = 7)	P-value
Treatment urogenital, n (%)			<0.0001^1^
No injury present	0 (0.0%)	5 (71.4%)	
Non-operative treatment	1 (100.0%)	0 (0.0%)	
Any intervention other than Foley	0 (0.0%)	2 (28.6%)	

**Table 14:** A comparison of treatment modalities for urogenital injuries according to Torode fracture classification for patients in the pelvic fracture group, represented as raw values and percentages.​​​​​​​ ^1^ Chi-square p-value.

	I (N = 68)	II (N = 11)	IIIA (N = 22)	IIIB (N = 30)	IV (N = 16)	P-value
Treatment urogenital, n (%)						<0.0001^1^
No injury present	67 (98.5%)	5 (45.5%)	17 (77.3%)	24 (80.0%)	7 (43.8%)	
Non-operative treatment	1 (1.5%)	5 (45.5%)	5 (22.7%)	6 (20.0%)	5 (31.3%)	
Any intervention other than Foley	0 (0.0%)	1 (9.1%)	0 (0.0%)	0 (0.0%)	4 (25.0%)	

According to Table 15, imaging modalities utilized differed significantly depending on fracture type (χ²(16) ≈ 72, p < 0.0001, Cramér’s V = 0.35). Pelvic ring fractures and acetabular fractures required additional imaging beyond X-ray more often than femur fractures, with computed tomography (CT) with contrast being utilized 36.2% (n = 54) and 42.3% (n = 11) of the time, respectively. The percentage of patients admitted to the ICU demonstrated a significant difference between fracture groups (χ²(2) ≈ 21, p < 0.0001, Cramér’s V = 0.26), indicating pelvic ring fractures had the highest rate (24.8%, n = 37) of ICU admission. The number of days spent in the ICU (p = 0.8661), total length of hospital stay (p = 0.0754), and percentage of patients requiring placement of a Foley catheter (p = 0.2677) did not show significant differences between fracture types.

**Table 15:** A comparison of secondary measures regarding patient care according to fracture diagnosis. Data represented includes imaging modalities used other than X-ray (i.e., computed tomography (CT), magnetic resonance imaging (MRI), ultrasound (US), cystogram, etc.), presence and length of intensive care unit (ICU) stay, length of hospital stay, and placement of a Foley catheter. Mean (standard deviation), median, and range values are provided for continuous data and raw values and percentages are provided for categorical data. ^1^ ANOVA F-test p-value. ^2^ Chi-square p-value. w/o: without; w/: with.

	Pelvic ring fracture	Femur fracture	Acetabular fracture or hip dislocation	P-value
	N = 149	N = 128	N = 26	
Imaging, n (%)				<0.0001^2^
No additional imaging	76 (51.0%)	104 (81.9%)	5 (19.2%)	
CT w/o contrast	7 (4.7%)	9 (7.1%)	7 (26.9%)	
CT w/ contrast	54 (36.2%)	8 (6.3%)	11 (42.3%)	
CT w/ and w/o contrast	2 (1.3%)	0 (0.0%)	0 (0.0%)	
Cystogram + CT	5 (3.4%)	0 (0.0%)	1 (3.8%)	
Ultrasound	0 (0.0%)	1 (0.8%)	1 (3.8%)	
MRI	1 (0.7%)	4 (3.1%)	0 (0.0%)	
Multiple	4 (2.7%)	1 (0.8%)	1 (3.8%)	
Missing	0	1	0	
ICU stay, n (%)				<0.0001^2^
No	112 (75.2%)	122 (95.3%)	23 (88.5%)	
Yes	37 (24.8%)	6 (4.7%)	3 (11.5%)	
ICU days				0.8661^1^
N	37	6	3	
Mean (SD)	9.6 (12.5)	10.5 (12.7)	6.0 (3.0)	
Median	4	4	6	
Range	1-54	1-32	3-9	
Hospital days				0.0754^1^
N	149	128	26	
Mean (SD)	5.4 (9.5)	3.4 (3.8)	4.0 (4.2)	
Median	2	3	2.5	
Range	0-60	0-32	1-20	
Foley catheter placement, n (%)				0.2677^2^
No	117 (79.1%)	110 (85.9%)	20 (76.9%)	
Yes	31 (20.9%)	18 (14.1%)	6 (23.1%)	
Missing	1	0	0	

## Discussion

The results of this study indicate pediatric patients with pelvic trauma primarily present with pelvic ring or femur fractures, are predominantly male, and vary in average age according to primary fracture diagnosis. Pelvic bony injuries were predominantly caused by falls or MVCs, with a significant majority of patients not experiencing any associated urogenital injury. For patients who experienced a pelvic ring fracture, most were classified as being of a milder severity according to both the Tile and Torode classifications. When patients with pelvic ring fractures demonstrated an associated urogenital injury, it was associated with a greater severity of injury according to both the Tile and Torode classifications. Regarding treatment, patients with pelvic ring fractures and acetabular fractures or hip dislocations more commonly received non-operative treatment, whereas those with femur fractures more often underwent surgery. Urogenital injuries in all groups were most treated non-operatively, with operative treatment being slightly more common in those with more severe pelvic ring fracture classifications.

Due to differences in anatomy and physiology, pelvic trauma in the pediatric population is a rare event and is less likely than in the adult population [14,15]. Studies show that pelvic fractures account for between 0.3% and 4% of all pediatric injuries, with an estimated incidence of around one per 100,000 children per year [14,16]. When present, they are often the result of high-energy trauma, putting the child at risk of increased morbidity and mortality [17]. Urogenital injuries present as one such complication, with one study indicating 17% of pelvic fractures involve a concomitant urogenital injury, with the incidence of urethral injuries estimated to be <1% to 7.5% [10,17]. In the adult population, pelvic trauma accounts for 2% to 8% of all adult skeletal trauma, with an estimated incidence of 20-37 per 100,000 adults per year [18-20]. Associated urogenital injuries are estimated to be involved in nearly 25% of adult cases [5].

Pediatric literature indicates children presenting with pelvic fractures are predominantly male, showing a ratio of 1.4:1.16. Our study showed an overall 2:1 male predominance, varying slightly depending on fracture type (Tables 1, 2). This stands in contrast to a large epidemiological study by Buller et al. in which nearly 70% of patients with pelvic ring fractures were females [18]. Less than 20% of that study population was under the age of 35 years, with 67% being 56 years or older [18]. This highlights important differences between the adult and pediatric populations. As reflected in this study, the literature regarding mechanisms of injury for pelvic trauma shows that MVCs and falls are the most common [18]. Compared to a literature-indicated average age of nine years old for pediatric patients experiencing pelvic trauma, our study showed an increased mean age of 11 years (Table 1) [16].

As may be expected, the incidence and severity of an associated urogenital injury are related to the severity of the pelvic fracture [5]. Pavelka et al. describe a study of 308 patients with pelvic fractures, classified as type A, B, or C based on increasing severity [9]. Of the 50 patients who experienced a urogenital tract injury, 61% were classified as type C [9]. This pattern was affirmed in our study, as fractures with a group IV Torode classification showed the highest rate of urogenital injuries at 56.2%, accounting for the greatest number of urogenital injuries per group (n = 9). Additional studies indicate the most important risk factor for urogenital injury is disruption of the pubic symphysis, with one study showing 42% of patients with disruption of the pubic symphysis had urological injuries [5]. Further, every one millimeter of diastasis of the pubic symphysis or dislocation of the inner-medial part of the pubic bone increases the risk of urethral injury by 10% [5,6]. With regard to fractures of the acetabulum, our study showed that no patients experienced associated urogenital injuries. While this is the predominant pattern seen in the adult population, one study indicated 2% of patients with acetabular fractures experienced complete rupture of the urethra, while 7% had partial urethral damage [5,6].

Urogenital injuries associated with pelvic trauma can occur anywhere along the urogenital tract, and include hematuria, urethral disruption, and bladder rupture [10]. Like other pediatric studies, our study showed hematuria was a commonly associated complication, while the 2% of patients in our study who experienced lower urinary tract injuries were similar to rates of 2.8% and <1%-5% cited in the literature [10,17]. In contrast, one adult study indicates urethral injuries may occur in up to 10% of cases, another cites an incidence of 14.6% for bladder or urethral injuries, while a third study cites rates of 5% for complete urethral rupture, 17% for partial urethral damage, and 7% for isolated damage to the urinary bladder [5,8,21]. In our study, urinary complications, where both urinary retention and incontinence were grouped as one problem, were quite common, but were rarely cited in the literature as an acute problem. It is possible these complications were related to factors surrounding treatment or the hospital stay, rather than due to direct injury. Overall, when pairing our study results alongside other pediatric studies, the overall incidence of lower urinary tract injury appears to be lower in pediatric patients than in adults [8].

Guillaume et al. and Gänsslen et al. both provide guidelines regarding the diagnosis and treatment of pelvic injuries in pediatric patients [2,16]. These guidelines are similar to those in adult orthopedic trauma care, such as evaluating for hemodynamic status, severity of bony injury, and concomitant injuries [2,14,16]. Imaging modalities include pelvic X-ray as the gold standard, with unstable fractures also requiring abdominal-pelvic CT [16]. Transcranial Doppler ultrasound, focused assessment with sonography in trauma (FAST), and a full-body CT scan are also recommended in the setting of high-energy trauma [2]. The recommendation of CT scan results from increased precision, as one study cites 30% of pelvic fractures identified on CT were not seen on initial plain X-rays [2]. In our study, 63 of 149 patients with pelvic ring fractures underwent some form of CT scan (Table 11). Surgical treatment depends on the hemodynamic stability of the patient, presence of additional injuries, and stability of the pelvis [2,16]. Treatment patterns in this current study followed such patterns, as 10.7% (n = 16) of patients with pelvic ring fractures underwent operative treatment (Table 8), a number mirroring the number of patients with a Torode IV fracture (n = 16) (Table 3).

The presence and treatment of urogenital injuries were not common in our study. As such, the discussion of diagnosis and treatment of urogenital injuries will primarily focus on current literature. Studies indicate the gold standard for diagnosis is retrograde urethrogram (RUG) [16,17,22-24]. Catheterization is controversial due to the potential of causing further injury, and it is recommended to wait for catheterization until RUG confirms an intact urethra [22,23]. Hagedorn et al. report RUG is not feasible in girls due to a shorter urethral length, thus recommending evaluation with cystoscopy and vaginoscopy under general anesthesia [17]. Definitive treatment of urogenital injuries is controversial. For urethral injuries, most studies recommend suprapubic catheter drainage with delayed repair, while a few others propose primary realignment [17,24]. The proposal for deferred repair is to avoid complications such as urethral strictures that commonly arise following early primary repair [17,24]. In the case of bladder rupture, extra-peritoneal ruptures may be managed non-operatively, while intra-peritoneal ruptures require primary surgical repair [16,21,22].

Regarding the long-term effects of pelvic trauma, a few studies describe residual pain, limping, scoliotic posture, or low back pain, and permanently associated urological or neurological damage as potential long-term sequelae of pelvic injuries [2,16]. These are reported in around 30% of patients with unstable pelvic fractures, with stable fractures rarely presenting with complications [2]. Specifics of long-term effects of urogenital injuries are less well-described for the pediatric population, but a greater understanding may be gleaned from adult studies. The previously mentioned study by Pavelka et al. reported an average follow-up time of 71 months [9]. A total of 50 patients sustained urogenital injuries, including 23 with urethral trauma and 18 with urinary bladder trauma. Patients with urethral trauma reported urinary incontinence, erectile dysfunction, and urethral stenosis, while those with bladder injury reported no residual complaints at one-year follow-up [9]. Rovere et al. describe a significant prevalence of sexual dysfunction following pelvic injuries that may be attributed to neurological and/or vascular injury [25]. These long-term sequelae can lead to decreased quality of life and a negative impact on patients’ mental health [9,25]. The impact of such complications in pediatric patients is less understood.

Limitations of this study include the retrospective design, variability of available data within patient charts, and difficulty controlling confounding factors. Due to the retrospective design, we were unable to establish causal relationships or evaluate long-term outcomes. Information regarding urogenital injuries was particularly difficult to assess due to a lack of charted details, leading to a small subset of data and difficulty in establishing meaningful relationships through statistical analysis. Additionally, as a small group of patient charts was inaccessible, it is possible that patients with more severe injuries were left out of the cohort. Last, identification and diagnosis of fractures were not performed by trained radiologists, presenting the potential for inaccurate fracture classifications.

## Conclusions

While existing studies provide important insight into pediatric pelvic trauma and its association with urogenital injuries, further research is needed to better define this relationship. Larger, multi-institutional cohorts would improve generalizability, while longitudinal studies could clarify long-term outcomes in this population. Although current diagnostic and treatment guidelines offer a strong foundation, continued investigation may help refine and standardize management strategies for pediatric patients.

Our findings highlight clinically meaningful differences between pediatric and adult populations, with pediatric patients demonstrating a lower overall incidence of urogenital injury and distinct injury patterns when present. Pediatric pelvic trauma occurs more frequently in males, and the risk of associated urogenital injury increases with fracture severity. These insights can aid both orthopedic surgeons and urologists in risk stratification and clinical decision-making. Based on these findings, we recommend early interdisciplinary communication to optimize management and improve the timeliness of care.
